# CSFCNet: Cascaded Spatial-Frequency Convolutional Network for Hyperspectral Image Classification

**DOI:** 10.3390/s26082325

**Published:** 2026-04-09

**Authors:** Feng Jiang, Xin Liu, Mingxuan Li, Ting Nie, Liang Huang

**Affiliations:** 1 Changchun Institute of Optics, Fine Mechanics and Physics, Chinese Academy of Sciences, Changchun 130033, China; jiangfeng23@mails.ucas.ac.cn (F.J.); ciompliuxin@ciomp.ac.cn (X.L.); limingxuan17@mails.ucas.ac.cn (M.L.); nieting@ciomp.ac.cn (T.N.); 2 University of Chinese Academy of Sciences, Beijing 100049, China

**Keywords:** hyperspectral image classification, Fourier transform, spatial convolution, local attention mechanism

## Abstract

CNNs can effectively extract features with low computational costs, achieving significant progress in hyperspectral image classification. However, due to the limited receptive field of CNNs, they have difficulty in capturing the multi-scale structural and global contextual information. Moreover, the class imbalance in hyperspectral images often causes the model to focus disproportionately on certain spectral bands, thereby reducing the average accuracy. To address these challenges, a method called the Cascaded Spatial-Frequency Convolutional Network (CSFCNet) was proposed for hyperspectral image classification. It integrates rich spatial-domain information and frequency-domain information by jointly modeling both domains. Specifically, a Dual Spatial Fourier Convolution (DSF-Conv) module was proposed to project feature maps into parallel spatial and frequency representations. In the Spatial pathway, input features are grouped and processed with multi-scale convolutions to extract hierarchical structures; in the Fourier pathway, frequency-domain convolutions can aggregate the global context. Subsequently, a group-cascaded structure connects the DSF-Conv modules with residual connections, alleviating the class imbalance problem by promoting more balanced contributions from different spectral components. Additionally, we introduce a Lightweight Local Attention module to enhance the feature discrimination. Furthermore, experiments on three datasets achieved competitive accuracies, demonstrating the effectiveness of CSFCNet. Ablation studies further verify the effectiveness of the core components within the network.

## 1. Introduction

Hyperspectral images (HSIs) are captured by imaging spectrometers across hundreds of contiguous spectral bands, forming three-dimensional data cubes rich in spatial–spectral information. Due to this rich information content, HSIs are widely utilized in fields such as geology and mineralogy [1,2], atmospheric science [3], and agriculture [4]. The primary objective of hyperspectral image classification (HSIC) is to accurately identify the category of each pixel and assign a corresponding unique label. Accurate pixel-level classification serves as a prerequisite for various downstream applications.

In recent years, deep learning (DL) has become the dominant paradigm in image processing, driven by its powerful hierarchical representation capabilities. Among various DL architectures, Convolutional Neural Networks (CNNs) [5] have gained widespread adoption by leveraging local connectivity [6] and weight sharing [7]. These structural properties facilitate the extraction of discriminative local spatial–spectral features while maintaining computational efficiency. As a result, CNN-based methods have become the mainstream approach for HSIC [8,9,10], demonstrating strong performance in modeling complex spectral and spatial dependencies in hyperspectral data.

Numerous CNN-based methods have been developed to further improve classification performance. For example, Roy et al. [11] proposed HybridSN, which fuses a spectral–spatial 3D-CNN layer with a spatial 2D-CNN layer to exploit joint spectral–spatial features. Subsequently, they introduced an approach utilizing selective 3D Res-convolution kernels to further strengthen these features [12] and later presented a weighted combination of bidirectional feature maps with morphological operators to significantly improve classification performance [13]. In another study, Zhai et al. [14] developed an effective 1D-CNN that iteratively explores local channel correlations to comprehensively utilize both shallow and deep features. Fu et al. [15] constructed a local–global gated CNN capable of filtering noise and extracting useful information from HSIs. Furthermore, Li et al. [16] proposed a dual single-channel (DSC) 3D convolution paired with depthwise convolution to fully extract spectral–spatial information. They also designed a channel and a layer-oriented network, which outperforms existing methods in classification accuracy with reduced computational cost.

Despite these advances, most CNN-based methods remain inherently limited by their local receptive fields, which restrict their ability to capture multi-scale structural and global contextual information [17]. Moreover, because HSIs contain hundreds of contiguous spectral bands, different features of classes are often unevenly distributed across them. Conventional spatial convolutions tend to focus on spectral bands with abundant training samples while underutilizing those associated with underrepresented classes [18]. Consequently, discriminative information in these bands is not effectively captured. This leads to poor classification performance for the corresponding land-cover categories and ultimately reduces the overall accuracy [19].

**Figure 1:**
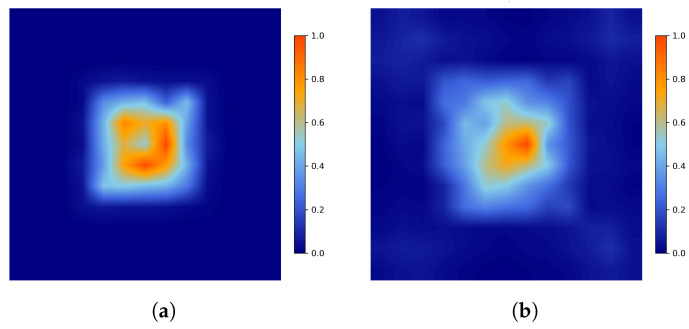
Receptive fields of a (**a**) spatial pathway and a (**b**) Fourier pathway.

To overcome these limitations, we introduce the Cascaded Spatial–Frequency Convolutional Network (CSFCNet) for hyperspectral image classification. CSFCNet integrates rich spatial-domain and frequency-domain information by jointly modeling both domains. At its core, a Dual Spatial–Fourier Convolution (DSF-Conv) module is designed to project feature maps into parallel spatial and frequency representations. In the spatial pathway, input features are grouped and processed using multi-scale convolutions to capture hierarchical structures. In the Fourier pathway, frequency-domain convolutions enable global context modeling by aggregating long-range dependencies. Figure 1 illustrates the differences in receptive fields between the proposed spatial and Fourier pathways. Furthermore, a group-cascaded structure connects the DSF-Conv modules with residual connections, alleviating the class imbalance problem by promoting more balanced contributions from different spectral components. In addition, a Lightweight Local Attention (LLA) module is incorporated to emphasize informative spatial regions, thereby improving feature discrimination.

The contributions of this article can be summarized as follows:

(1) To address the limitations of CNNs in capturing multi-scale structures and global contextual information, we propose a Cascaded Spatial–Frequency Convolutional Network (CSFCNet) that jointly models spatial- and frequency-domain representations for hyperspectral image classification;
(2) To effectively integrate local and global features, we design a Dual Spatial–Fourier Convolution (DSF-Conv) module that constructs parallel spatial and frequency pathways, enabling the joint modeling of hierarchical structures and long-range dependencies;
(3) To enhance feature discrimination, we introduce a Lightweight Local Attention (LLA) module that adaptively emphasizes informative spatial regions;
(4) Extensive experiments on three benchmark datasets demonstrate that CSFCNet achieves competitive accuracies, validating its robust performance. Furthermore, comprehensive ablation studies verify the specific contributions of the core components, ensuring the effectiveness of the proposed architectural designs.

The remainder of this paper is organized as follows: Section 2 reviews existing hyperspectral image classification methods, especially frequency-domain methods. Section 3 details the proposed method. Section 4 validates its effectiveness through comprehensive experiments. Finally, Section 5 presents the conclusions of this paper.

## 2. Related Works

### 2.1. Hyperspectral Image Classification

Hyperspectral image classification methods have evolved from traditional approaches to deep learning techniques. Traditional methods typically rely on handcrafted feature descriptors. For example, Yang et al. [20] proposed a collaborative representation using local adaptive dictionaries. It reduces the adverse effects of irrelevant pixels. Fang et al. [21] took a different route. They encoded inter-band relationships through local covariance matrices and fed these into support vector machines for discrimination. Composite kernels have also been exploited to fuse spatial and spectral signals [22]. Li et al. [23] pursued multi-feature combination learning for high-resolution scene parsing.

However, these machine-learning pipelines often falter when modeling intricate spectral–spatial couplings. Deep learning has emerged as the preferred alternative. Convolutional Neural Networks, in particular, have gained wide adoption. Their strength lies in automatically distilling hierarchical features directly from raw data. This bypasses the need for laborious manual design while providing superior representational power.

For instance, 3D convolutional networks [24] capture sophisticated features via simultaneous processing in both the spectral and spatial dimensions. While they can extract spatial and spectral information effectively, they have high computational costs and are prone to overfitting. To address these limitations, some studies have proposed hybrid convolutional networks that combine two-dimensional convolutions (for spatial feature extraction) with one-dimensional convolutions (for spectral feature extraction). Although these methods have improved classification performance to some extent, they still suffer from a limited receptive field, which restricts their ability to capture global information. In response to this, researchers have proposed various improvement methods. The primary approaches include using dilated convolution, attention mechanisms, and large-kernel convolution.

Dilated convolution expands the receptive field by inserting intervals within the convolution kernel. Hu et al. [25] combined one-dimensional dilated convolution to extract spectral features with two-dimensional dilated convolution to extract spatial features, enabling the modeling of long-sequence information in hyperspectral images. Li et al. [26] achieved this by fusing local convolution with mixed dilated convolution, which not only closely connects local pixels but also enhances the flexibility and expressiveness of the convolutional layer. Although dilated convolution can capture larger-scale contextual information without adding extra parameters, a high dilation rate may cause information loss, especially when processing high-frequency details. Moreover, its receptive field size is fixed and cannot adaptively adjust to capture multi-scale features.

The attention mechanism enhances important features through weighting, thereby improving feature discriminability. Mou et al. [27] employed gated mechanisms and self-attention to recalibrate the importance of different spectral bands, selectively emphasizing information-rich bands. By integrating SplitConv to halve input channels, the self-attention mechanism in Xia et al.’s work [28] dynamically recalibrates feature weights, effectively pruning redundant information for a lightweight architecture. However, the attention mechanism significantly increases the model’s parameters, leading to higher computational costs, and primarily focuses on feature weighting without fundamentally addressing the limited-receptive-field problem. Furthermore, the attention mechanism tends to assign greater weights to positions with a large number of samples and spectral bands, resulting in decreased classification accuracy for local objects with few samples.

Another strategy to expand the receptive field is the use of large-kernel convolutions, which aim to mimic the global connectivity of transformers. For instance, Sun et al. [29] proposed a large-kernel spectral–spatial attention network (LKSSAN), which utilizes large-kernel attention (LKA) to capture long-range 3-D spectral–spatial properties. By decomposing a large-scale convolution into spatial local, spatial long-range, and channel-wise components, it effectively exploits discriminative features while maintaining the volumetric structure of HSIs. Similarly, Liu et al. [30] developed a multi-scale CNN architecture that leverages asymmetric convolutions from small to large kernels. This approach employs asymmetric kernels to decompose standard square large kernels, enabling the extraction of multi-scale spatial features across various ranges while significantly reducing parameter redundancy. While large kernels effectively enlarge the effective receptive field, they often lead to significant increases in parameter redundancy and computational complexity [31]. Moreover, simply increasing the kernel size lacks the adaptive frequency-domain awareness required to process the complex spectral signatures found in hyperspectral data.

### 2.2. Frequency-Domain Convolutional Method

In recent years, frequency-domain convolution has attracted increasing attention in deep learning. Among its variants, Fourier convolution and wavelet convolution have shown notable progress.

The Fourier convolution process manages signals by transforming them from the time domain to the frequency domain, leveraging the global characteristics of the frequency domain to effectively capture periodic patterns and global features of the signals. Wu et al. [32] demonstrate that the Fourier spectrum is mathematically equivalent to the quadratic-form energy redistribution performed by multi-head attention. For example, Suvorov et al. [33] proposed a resolution-robust large-mask method based on Fourier convolution for image processing. Additionally, Yi et al. [34] introduced a frequency-domain module to capture the dynamic and complex periodic patterns of time series data. In recent years, Fourier convolution has been incorporated into HSI classification to overcome the limited receptive field of traditional spectral–spatial convolution. Shi et al. [35] proposed F3Net (Fast Fourier Filter Network), which directly designs learnable filters in the discrete Fourier domain and suppresses redundant frequency components through a frequency-domain gating mechanism. Pan et al. [36] further developed FSFF-Net, explicitly fusing dual-stream features from the spatial and frequency domains and achieving the adaptive weighting of heterogeneous features through cross-covariance attention, significantly enhancing robustness in small-sample scenarios. Moreover, He et al. [37] proposed a decoupled image-frequency spectral–spatial framework that uses two-dimensional Fourier convolution to capture spatial structures along the height and width dimensions, followed by one-dimensional Fourier convolution along the spectral dimension to model the global spectral response, achieving end-to-end dimension-free classification. These studies demonstrate the unique advantages of Fourier convolution.

Despite the remarkable progress made in techniques based on the Fourier transform and their convolutional networks, certain limitations remain in their universal application. The global nature of Fourier convolution, while effective for capturing periodic patterns, causes considerable deficiencies in representing local aperiodic elements and spatially heterogeneous characteristics. As a result, this restriction presents itself with particular severity in hyperspectral images, and localized spectral–spatial variations constitute discriminative information that is critical to classification [38]. Convolutions in the spatial domain, thus, come to the fore as functionally complementary mechanisms capable of addressing these representational inadequacies. Moreover, Fourier-transform operations come at a considerable computational cost and are highly unscalable with large data [39]. Carrying out forward and backward transformations at each convolutional layer significantly increases the overall computational cost, thus limiting their applicability in low-resource settings [40].

Wavelet convolution combines temporal and frequency-domain information, resulting in a multi-resolution representation of signals that better captures local features and multi-scale patterns. The theory proposed by Mallat on wavelet analysis has provided the basis of wavelet convolution [41]. In recent work, Finder et al. [42] proposed a wavelet-based convolution to enlarge the receptive field of CNNs. Furthermore, Wen et al. [43] applied the maximal overlap discrete wavelet transform to decompose various wavelet coefficients on each level in order to detect the singular periodicities on each level. These studies prove that the multi-scale feature extraction and local feature capture ability of the wavelet convolution is quite strong. Ahmad et al. [44] proposed an architecture that employs wavelet-reversible downsampling in conjunction with the transformer self-attention mechanism to enhance the multi-scale representation of spectral–spatial features of hyperspectral images without losing any information.

However, wavelet convolution has several limitations. Its performance heavily depends on the choices of the wavelet basis and decomposition level, requiring careful tuning. Multi-level decomposition also incurs high computational costs, limiting scalability for large datasets. Furthermore, the resulting wavelet coefficients often contain redundant information, which may introduce noise and degrade model performance [45,46].

## 3. Proposed Method

### 3.1. Overall Framework

**Figure 2:**
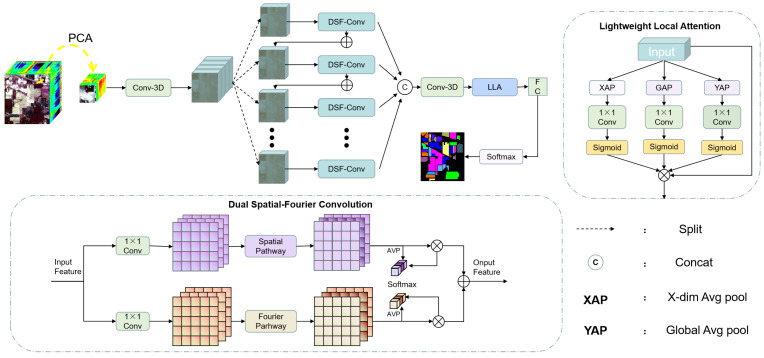
Overall framework of the proposed CSFCNet.

The overall framework of the CSFCNet is shown in Figure 2. Specifically, CSFCNet consists of group-cascaded Dual Spatial–Fourier Convolution modules, a Lightweight Local Attention module, and some convolutional layers. CSFCNet consists of three stages: The first stage involves sample extraction from HSI data; in the second stage, cascaded DSF-Conv modules are employed to extract features from hyperspectral images. The third stage, referred to as the HSI classification stage, utilizes a Lightweight Local Attention module and a fully connected layer to extract key spatial–spectral information and generate the classification output for hyperspectral images.

Sample extraction: In this stage, the dimension of the image is reduced to *L* using principal component analysis (PCA) and then a data cube block with spatial dimensions *S* and spectral bands *L* is extracted as a sample, where the block size is defined as S×S×L. Each cube is centered at a target pixel, and its label is assigned according to the central pixel. The spatial size (*S*) is set at an odd number to ensure a well-defined center. The extracted cube is then fed into a 3D-convolutional layer to compress spectral information and obtain initial feature representations.

Cascaded DSF-Conv modules: This stage employs *N* DSF-Conv modules in a cascaded manner to progressively extract spatial–spectral features. Each DSF-Conv module consists of three components: a spatial pathway, a Fourier pathway, and a channel selection unit. Residual connections are introduced to facilitate feature reuse and stabilize training. Specifically, the spatial pathway focuses on capturing local and multi-scale structural information, while the Fourier pathway models global dependencies through frequency-domain representations. The channel selection unit adaptively fuses spatial and frequency features to retain the most discriminative information.

HSI classification stage: A Lightweight Local Attention module assigns higher weights to the key local regions, thereby enhancing the proportion of these regional features in the global feature representation and integrating local texture features with deep features. Finally, the feature maps are mapped to each category through FC and softmax, completing the hyperspectral image classification.

### 3.2. Dual Spatial-Fourier Convolution Module

Figure 2 presents the designed DSF-Conv module, which consists of three core components: the spatial pathway, the Fourier pathway, and the channel selection unit. Specifically, the DSF-Conv generates spatially refined features ($Y^{s}$) and frequency-refined features ($Y^{f}$) by means of the spatial pathway and Fourier pathway, respectively. After that, the representative features (*Y*) are preserved via the operation implemented by the channel selection unit.

**Figure 3:**
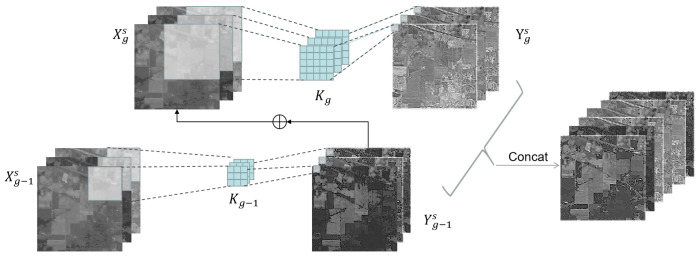
The structure of the spatial pathway.

Within the spatial pathway, the channels of the feature maps are initially split, followed by convolution kernels of different sizes to capture multi-scale features. Furthermore, hierarchical residual connections are established among different kernels to progressively enlarge the receptive field of each convolutional layer. As shown in Figure 3, we evenly split the spatial part ($X^{s}$) into *G* feature map groups ($X_{g}^{s}$), where g∈{1,2,…,G}. Each group ($X_{g}^{s}$) has the same spatial dimension as that of $X^{s}$ but has only a 1/G channel length. For each $X_{g}^{s}$, there exists a corresponding convolution kernel with a size of $k_{g}$, denoted as $K_{g}\in R^{C\times k_{g}\times k_{g}\times C}$. For simplicity, we omit the summation operations of each dimension in the convolution. Therefore, the output ($Y_{g}^{s}$) of the $K_{g}$ can be represented as follows:

(1) $$Y_{g}^{s}=\left\{\begin{array}{cc} X_{g}^{s}*K_{g}, & g=1\\ \left(X_{g}^{s}+Y_{g-1}^{s}\right)*K_{g}, & 1<g\leq G \end{array}\right.$$

Specifically, for the *g*th group of spatial convolution kernels, the expansion of the kernel size ($k_{g}$) and the receptive field ($RF_{g}$) are defined as follows:

(2) $$\begin{array}{cc} k_{g+1} & =k_{g}+2\\ RF_{g+1} & =RF_{g}+\left(k_{g+1}-1\right) \end{array}$$

As each convolution ($k_{g}$) could receive feature information from all the feature groups, the output ($Y_{g}^{s}$) exhibits a larger receptive field than the preceding group. This multi-scale design enables the model to capture richer contextual information, improving feature representation. To better fuse information at different scales, all the groups are concatenated along the channel dimension and then passed through a 1 × 1 convolution expressed as follows:

(3) $$Y^{s}=\sum_{g=1}^{G}\left[Y_{1}^{s},Y_{2}^{s},\ldots ,Y_{g}^{s}\right]*k_{1\times 1}$$

The variable *g* serves as a regulatory parameter that governs the dimensionality of the scale. An incremented value of *g* facilitates the convolutional layer’s ability to concurrently process features across a spectrum of granularities. This approach is instrumental in mitigating the presence of redundant features and bolstering the capacity for robust feature representation. The resultant spatially refined features are denoted as $Y^{s}$.

**Figure 4:**
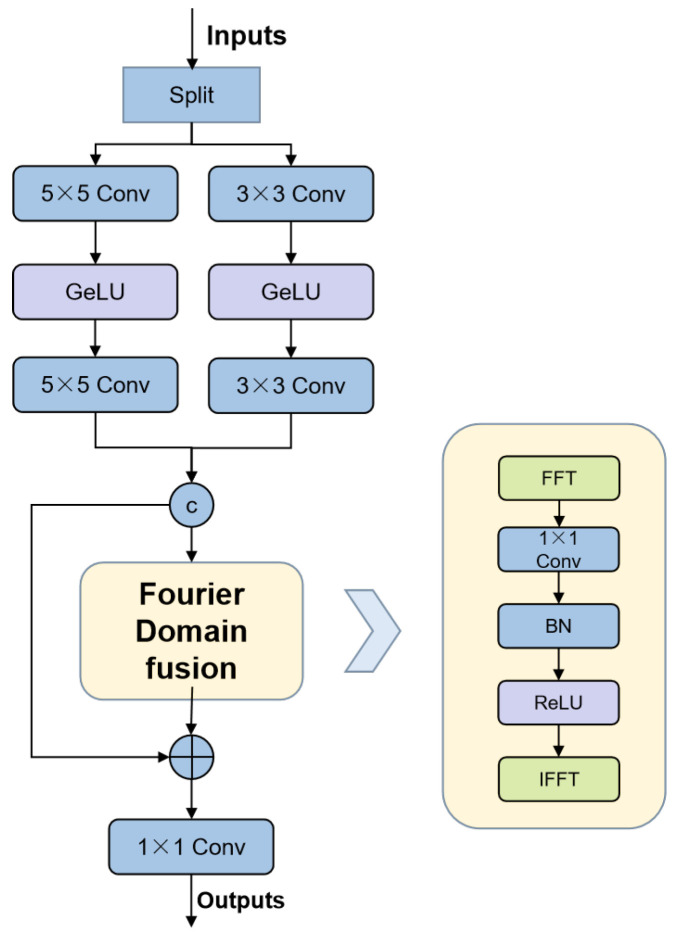
The structure of the Fourier pathway.

Our Fourier pathway is illustrated in Figure 4. To capture global contextual dependencies, we transform spatial features into the frequency domain using the Discrete Fourier Transform (DFT). Given an input feature map ($X^{f}$), the 2D DFT is defined as follows:

(4) $$F\left(u,v\right)=\frac{1}{\sqrt{HW}}\sum_{h=0}^{H-1}\sum_{w=0}^{W-1}X^{f}\left(h,w\right)e^{-j2\pi \left(\frac{hu}{H}+\frac{wv}{W}\right)}$$

where (u,v) denotes frequency coordinates, and *H* and *W* denote the height and width of the spatial dimensions, respectively. Since each frequency component aggregates information from all the spatial locations, the Fourier representation naturally captures global contextual dependencies.

To enable learnable feature transformation in the frequency domain, the complex-valued spectrum is decomposed into real and imaginary components and jointly processed via a convolution as follows:

(5) F=ℜ(F)+jℑ(F)

(6) $$F_{r}=\sigma \left(\mathrm{BN}\left(ℜ,ℑ\right)*k_{1\times 1}\right)$$

Finally, the frequency feature is transformed back to the spatial domain and fused with the input via a residual connection as follows:

(7) $$Y^{f}=\sigma \left(\mathrm{BN}\left(X^{f}+F^{-1}\left(F_{r}\right)\right)\right)$$

This allows the Fourier pathway to capture global dependencies while complementing the spatial pathway’s local focus.

Having obtained the spatial-domain features ($Y^{s}$) and the frequency-domain features ($Y^{f}$), a fusion method is needed to adaptively select more discriminative features.

Specifically, we initially employ global average pooling (GAP) to aggregate global spatial and frequency information via channel-wise statistics ($S^{n}\in R^{C\times 1\times 1}$) with n∈{s,f}, which can be represented as follows:

(8) $$S^{n}=GAP\left(Y^{n}\right)=\frac{1}{H\times W}\sum_{i=0}^{H-1}\sum_{j=0}^{W-1}Y_{i,j}^{n}$$

Next, $S^{s}$ and $S^{f}$ are stacked together, and a channel-wise soft attention operation is utilized to derive feature selectivity weights ($\gamma ,\beta \in R^{C}$), which definitions are given below:

(9) $$\gamma =\frac{e^{S^{s}}}{e^{S^{s}}+e^{S^{f}}},\;\beta =\frac{e^{S^{f}}}{e^{S^{s}}+e^{S^{f}}}$$

Finally, under the guidance of the feature selectivity weights (γ and β), the ultimate output (*Y*) can be yielded by integrating $Y^{s}$ and $Y^{f}$ as follows:

(10) $$Y=\gamma Y^{s}+\beta Y^{f}$$

Overall, the proposed DSF-Conv can extract informative and discriminative object features in hyperspectral images.

### 3.3. Group Cascade Structure

For the design of the group cascade structure, we adapt a group cascade structure. The reason for grouping along the channel dimension is that adjacent spectral bands typically contain similar spatial features. The goal of fusing information across spectral bands is to achieve complementary information between different bands. This grouping strategy ensures that the features from one spectral band are incorporated into those of more distant bands, facilitating more effective cross-band interaction. Assuming the input feature $x\in R^{C\times H\times W}$, group the input tensor (*x*) along the channel dimension into *N* groups, with each group having $\frac{C}{N}$ channels as follows:

(11) $$x=\left[x_{1},x_{2},\ldots ,x_{N}\right],\;C_{N}=\frac{C}{N}$$

where *C* represents the number of channels in the input tensor. The output of the previous group is element-wise added to the input of the next group after passing through the Dual Spatial-Fourier Convolution module, forming a residual connection. The output of the group cascade can be expressed as follows:

(12) $$x_{\mathrm{output}}=\mathrm{Cat}\left(x_{1}^{\prime },x_{2}^{\prime },\ldots ,x_{N}^{\prime }\right)+x$$

where Cat represents the operation of concatenation.

### 3.4. Lightweight Local Attention Module

In comparison to traditional attention mechanisms, which rely on heavy computations, our method reduces the complexity of 3D attention to 1D attention in three orthogonal directions with a similar diverse power.

The LLA module can be formulated as follows. Given an input feature map ($X\in R^{C\times H\times W}$), where *C*, *H*, and *W* denote the number of channels, height, and width, respectively, the attention mechanism is computed as follows:

(13) $$Y=X⊙A_{h}⊙A_{w}$$

where ⊙ represents element-wise multiplication, and $A_{h}\in R^{C\times H\times 1}$, $A_{w}\in R^{C\times 1\times W}$ are the attention maps for the height and width dimensions, respectively. The height attention map ($A_{h}$) is computed as follows:

(14) $$A_{h}=\sigma \left(\mathrm{GN}\left({\mathrm{DWConv}}^{1D}\left({\bar{X}}_{h}\right)\right)\right)$$

where ${\bar{X}}_{h}$ is the height-wise average pooling, ${\mathrm{DWConv}}^{1D}$ denotes a 1D depthwise convolution with kernel size *k*, GN represents group normalization with 16 groups, and σ is the sigmoid activation function.

Similarly, the width attention map ($A_{w}$) is computed as follows:

(15) $$A_{w}=\sigma \left(\mathrm{GN}\left({\mathrm{DWConv}}^{1D}\left({\bar{X}}_{w}\right)\right)\right)$$

where ${\bar{X}}_{w}$ is the width-wise average pooling.

To enhance the understanding of the network structure, we outline the sequential steps of the model in Algorithm 1.
**Algorithm 1** Inference Process of CSFCNet**Require:** The original HSIs.
**Ensure:** Predicted labels of all the pixels.
1: **Initialization:**2: Perform PCA to reduce the dimension;3: Apply a 3D convolution layer with a kernel size of 3×3×3;4: Employ *N* cascaded DSF-Conv modules to progressively extract features;5: Apply a Lightweight Local Attention module to enhance spatial-channel features;6: Perform a 3×3 convolution layer;7: Apply global average pooling and layer normalization to obtain feature vectors;8: Perform the linear layer to obtain classification results.


## 4. Experiments

In this section, three widely used hyperspectral datasets and the experimental settings are first introduced. Subsequently, the proposed method is compared with eight representative models to demonstrate its effectiveness. Finally, ablation studies are conducted to analyze the contribution of each component in the network. To minimize the experimental variability, all the results are obtained by conducting five independent experiments.

### 4.1. Experimental Setting

#### 4.1.1. HSI Classification Datasets

To comprehensively evaluate the performance of the proposed method, experiments are conducted on three widely used hyperspectral benchmark datasets: Indian Pines (IP), Salinas (SA), and WHU-Hi-Honghu (HH).

Indian Pines: The Indian Pines dataset [47] was captured by the Airborne Visible Infrared Imaging Spectrometer (AVIRIS) in 1992 for an Indian Pines area in Indiana, USA. The original image has 220 bands, with wavelengths ranging from 0.4 to 2.5 μm. After removing the water absorption band, the remaining 200 bands were finally used as the research object. The data size is 145 × 145. There are a total of 10,249 land-cover pixels, representing 16 ground object categories. Detailed information on the Indian Pines dataset used in our experiments is presented in Table 1.

Salinas: The Salinas dataset [48] was acquired by the Airborne Visible Infrared Imaging Spectrometer (AVIRIS) in 1998 over the Salinas Valley in California, USA. The original image consists of 224 bands, with wavelengths ranging from 0.4 to 2.5 μm. After removing 20 water absorption bands, 204 effective bands remained. The image size is 512 × 217 pixels, with a spatial resolution of 3.7 m, and the dataset contains 54,129 ground object pixels corresponding to 16 ground object categories. Detailed information on the Salinas dataset used in our experiments is presented in Table 2.

WHU-Hi-HongHu: The WHU-Hi-HongHu dataset [49] was collected by Wuhan University in November 2017 using the Headwall Nano-Hyperspec sensor in the HongHu Wetland in Hubei Province, China. The original images contain 270 bands, with wavelengths ranging from 400 to 1000 nm; the image size is 940 × 475 pixels, with a spatial resolution of 0.043 m, and there are a total of 386,693 ground object pixels, corresponding to 22 land-cover categories. Detailed information on the HH dataset used in our experiments is presented in Table 3.

#### 4.1.2. Configuration

All the experiments were conducted on a server equipped with an Intel Xeon Platinum 8352V CPU (Intel, Santa Clara, CA, USA), 512 GB of RAM, and an NVIDIA RTX 4090 GPU (Nvidia, Santa Clara, CA, USA). All the models were implemented using PyTorch 2.1.0, and to reduce randomness, all the experiments are repeated five times, and the average results are reported.

The Adam optimizer was adopted, with an initial learning rate of $1\times 10^{-3}$, and the batch size was set at 64. The maximum number of training epochs was set at 150, 100, and 100 for the Indian Pines (IP), Salinas (SA), and WHU-Hi-Honghu (HH) datasets, respectively, considering their differences in data scale and complexity.

#### 4.1.3. Evaluation Metrics

To quantitatively evaluate the classification performance, three widely used metrics in hyperspectral image classification are adopted, including Overall Accuracy (OA), Average Accuracy (AA), and the Kappa coefficient (κ) [50]. These metrics are all derived from the confusion matrix [51].

OA measures the proportion of correctly classified samples over all the test samples. AA computes the average of class-wise accuracies, which provide a more balanced evaluation under class-imbalance conditions. The Kappa coefficient evaluates the agreement between the predicted labels and the ground truth while accounting for chance agreement.

### 4.2. Contrast Experiment

#### 4.2.1. Comparison with Other Outstanding Networks

In order to verify the superiority of the method we proposed, we compare CSFCNet with traditional algorithms and the most widely used and advanced algorithms. Eight HSI classification methods are selected for comparison, including one traditional machine-learning method (SVM [52]), three CNN-based methods (3DCNN [24], HybridSN [11], and CLOLN [16]), one frequency-domain-based method (HyperFKAN [53]), as well as three attention-based methods (GAHT [54], SSFTT [55], and CASANet [19]).

#### 4.2.2. Implementation Details

For all the deep-learning-based methods, we strictly followed the architectural configurations and hyperparameters reported in their original publications, including network depths, kernel sizes, and optimizer settings. The only modification was setting the input patch size at 13×13 pixels to match our CSFCNet, ensuring consistent spatial context across all the methods. The SVM was applied using only spectral information with 1×1 pixel inputs. We employed the RBF kernel with default parameters. All the methods were implemented in PyTorch and trained from scratch without pretrained weights. The same training split was used to ensure fairness.

#### 4.2.3. Quantitative Analysis

The detailed class-wise accuracies, Overall Accuracy (OA), Average Accuracy (AA), and Kappa coefficients are reported in Table 4, Table 5, and Table 6, respectively. The best results in each category are highlighted in bold.

The IP dataset is characterized by class imbalance and limited training samples. CSFCNet shows stable performance in this dataset, as shown in Table 4. The Overall Accuracy of CSFCNet (97.09%) is comparable to that of CSCANet (97.17%). However, CSFCNet achieves a highest Average Accuracy of 95.61%. This suggests that CSFCNet provides more balanced performance across classes. The improvement is more evident in minority classes. For example, for Grass-pasture-mowed (Class 7) and Oats (Class 9), CSFCNet achieves 99.08% and 94.74%, which are higher than the second-best results. Compared with CNN-based methods that focus on local features and transformer-based methods that rely on global attention, CSFCNet combines spatial and frequency representations. This helps to improve feature discrimination under limited samples. In addition, CSFCNet achieves the lowest OA standard deviation (±0.20%), indicating stable performance.

The SA dataset features high image quality and low classification difficulty. It can reflect the model’s classification performance under ideal conditions, as shown in Table 5. The superiority of CSFCNet is further consolidated, where it secures the highest performance across all the key metrics. CSFCNet attains an OA of 99.21% and an AA of 99.27%, outperforming the second-best methods (GAHT and HyperFKAN). And the Kappa coefficient of 99.12% further confirms the high consistency between the predicted maps and the ground truth.

The HH dataset is more complex, with diverse land-cover types and higher intra-class variability. CSFCNet still achieves competitive performance in this dataset, as shown in Table 6. It achieves an OA of 98.20% and the highest AA of 96.06%. In Broad bean (Class 21) and Tree (Class 22), CSFCNet achieves 94.77% and 97.92%, respectively. These results indicate better discrimination between similar classes. Compared with CNN-based and transformer-based methods, CSFCNet balances local detail extraction and global context modeling. The OA standard deviation is ±0.07%. This indicates stable performance across runs.

#### 4.2.4. Qualitative Analysis

**Figure 5:**
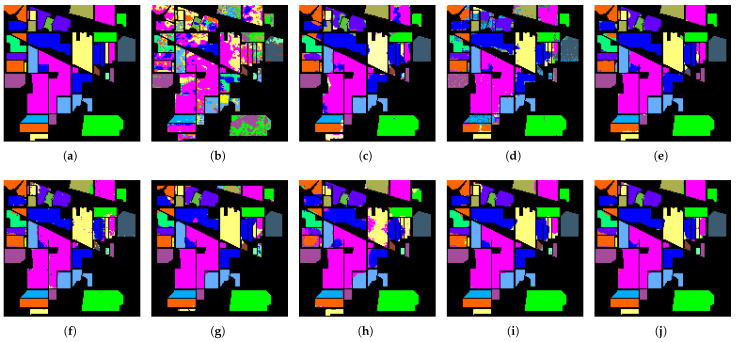
The classification maps of different networks in the IP dataset: (**a**) ground truth; (**b**) SVM; (**c**) 3DCNN; (**d**) HybridSN; (**e**) GAHT; (**f**) SSFTT; (**g**) HyperFKAN; (**h**) CLOLN; (**i**) CASANet; (**j**) proposed.

**Figure 6:**
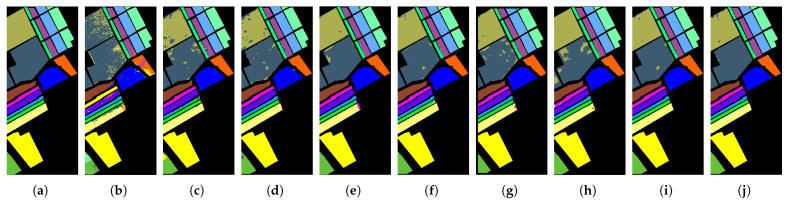
The classification maps of different networks in the SA dataset: (**a**) ground truth; (**b**) SVM; (**c**) 3DCNN; (**d**) HybridSN; (**e**) GAHT; (**f**) SSFTT; (**g**) HyperFKAN; (**h**) CLOLN; (**i**) CASANet; (**j**) proposed.

**Figure 7:**
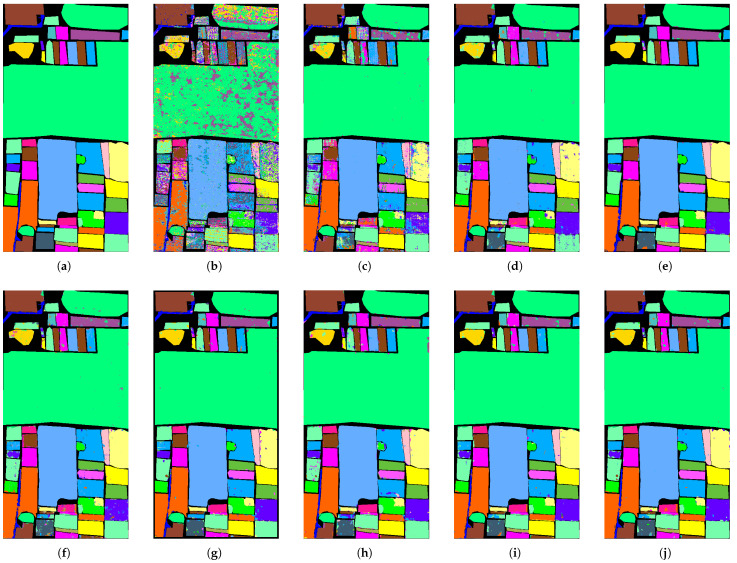
The classification maps of different networks in the HH dataset: (**a**) ground truth; (**b**) SVM; (**c**) 3DCNN; (**d**) HybridperSN; (**e**) GAHT; (**f**) SSFTT; (**g**) HyperFKAN; (**h**) CLOLN; (**i**) CASANet; (**j**) proposed.

Figure 5, Figure 6 and Figure 7 present the classification maps in the IP, SA, and HH datasets, which exhibit different spatial structures and levels of complexity. Overall, CNN-based methods, such as SVM, 3DCNN, and HybridSN, suffer from varying degrees of salt-and-pepper noise, where isolated misclassified pixels appear within homogeneous regions. This noise disrupts regional connectivity and reduces the spatial consistency, especially in the IP dataset with limited training samples. Although the effect is less severe in SA and HH, scattered artifacts can still be observed, indicating that CNN-based approaches remain sensitive to local noise.

In contrast, CSFCNet produces more homogeneous and spatially coherent regions across all the datasets. Large areas in SA and structured regions in HH remain compact, while fragmented regions in IP are significantly reduced. This improvement suggests enhanced regional connectivity and robustness, which can be attributed to the Fourier branch that captures global dependencies and suppresses noise within homogeneous areas.

From the boundary perspective, transformer-based methods, such as GAHT and SSFTT, tend to overemphasize global contextual information, which may lead to over-smoothed transitions between classes. This effect is more evident in complex scenes, such as urban regions in HH and fine-grained agricultural boundaries in SA, where edge details become less distinct. In comparison, CSFCNet preserves clearer and more continuous boundaries that better align with the ground truth. This is enabled by the dual-branch design, where spatial features retain high-frequency details and frequency features provide complementary global guidance.

For minority classes, CSFCNet maintains more complete and coherent structures across datasets. Small or scattered categories, such as “Oats” and “Stone-Steel-Towers” in IP and rare classes in HH, are clearly identified with reduced fragmentation. This indicates that the proposed cascaded structure enhances the representation of limited samples and helps to preserve discriminative features during classification.

#### 4.2.5. Sensitivity to the Training Sample Size

**Figure 8:**
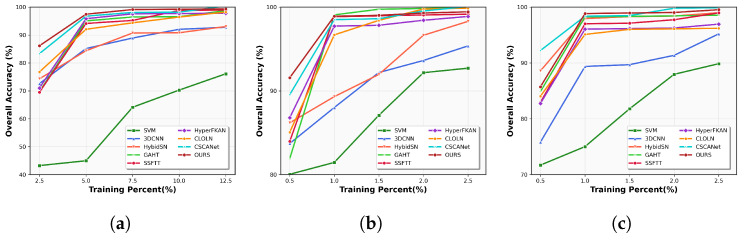
OAs for different proportions of training samples in the three datasets: (**a**) IP, (**b**) SA, and (**c**) HH, respectively.

To further verify the effectiveness of CSFCNet, the OA of the eight different algorithms was verified using different proportions of training samples, as shown in Figure 8. The training samples for the IP dataset were taken as 2.5%, 5%, 7.5%, and 10% of the dataset, and the training samples for the SA and HH datasets were taken as 0.5%, 1%, 1.5%, 2.0%, and 2.5%. From Figure 8, it can be seen that as the number of training samples increases, the model is more adequately trained, and the OA gradually improves. Also, among all the algorithms, CSFCNet shows the best classification performance, with OA steadily improving as the proportion of samples increases.

#### 4.2.6. Confusion Matrices

To further investigate the classification behavior in minority classes and verify whether the high Average Accuracy (AA) stems from genuine recognition rather than over-classification, we constructed precision-oriented confusion matrices for CSFCNet in all three datasets. Unlike standard row-normalized matrices that highlight recall, we employed column normalization to visualize precision. In these matrices, each column sums to 100%, representing the proportion of true classes among all the pixels predicted as a specific category.

**Figure 9:**
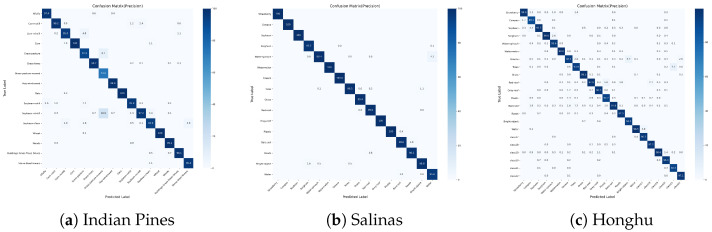
Column-normalized confusion matrices for CSFCNet in (**a**) IP, (**b**) SA, and (**c**) HH datasets; the numbers represent the accuracy.

As shown in Figure 9, the precision values for minority classes confirm that CSFCNet does not achieve high AAs by simply over-predicting rare classes. Instead, the model effectively learns discriminative features so that pixels classified as minority classes are highly reliable.

### 4.3. Ablation Study

To further explore and validate the contributions of different modules of the proposed CSFCNet model, ablation experiments are conducted on three difficult datasets, IP, SA, and HongHu, to analyze the effect of each component, including DSF-Conv, Group cascaded, and LLA modules. It is worth noting that when conducting the ablation study of the DSF-Conv module, we replaced it with a 3D-CNN. When we conducted the ablation of the group cascade structure, we replaced it with a DSF-Conv module with more channels.

The experimental results are presented in Table 7.

The removal of the DSF-Conv module resulted in a notable decline in the classification performance. Specifically, the overall accuracy dropped from 97.09% to 92.71%. Similarly, the same phenomenon was also observed in the other two datasets. These results underscore the critical role of the DSF-Conv module in enhancing the classification accuracy and robustness of the model.

The efficacy of the group cascade structure is verified through experiments on various hyperspectral image datasets. As witnessed in the above results, the group cascade architecture greatly enhances the network’s classification performance. For example, the group cascade structure can help to achieve an overall accuracy of 97.09%, an average accuracy of 96.63%, and a Kappa value of 97.23 in the Indian Pines dataset. In the absence of this structure, however, the performance suffers severely, and the OA falls down to 96.23%. Experiments on various hyperspectral image datasets validate the effectiveness of the LLA module. According to the results, the classification performance of the network improves significantly due to the LLA module. For example, the LLA module results in an overall accuracy value of 97.09% in the Indian Pines dataset. On the contrary, omitting this mechanism results in a marked dip in performance.

To summarize, our proposed module is more efficient than the standard convolution layer, having a significantly lower number of parameters while classifying images more accurately.

### 4.4. Parameter Analysis

#### 4.4.1. Using PCA to Reduce the Dimension

Due to the high dimensionality of hyperspectral images, principal component analysis (PCA) is adopted to reduce spectral redundancy and computational cost, following the common practice in HSIC. We compare the performances with and without PCA, where the number of spectral bands is reduced to 30. As shown in Table 8, applying PCA leads to significant improvements across all the evaluation metrics in both datasets. Specifically, OA, AA, and κ are consistently improved, while the standard deviations are reduced, indicating more stable performances. This suggests that PCA effectively removes redundant spectral information and preserves the most discriminative components, thereby facilitating more robust feature learning.

#### 4.4.2. Setting the Number of Groups in DSF-Conv

To investigate the influence of the group number in the DSF-Conv module, we conducted experiments on the IP dataset with group sizes set at {2,4,8, and 16}. The results are summarized in Table 9. The model achieves the optimal performance when the number of groups is set at 4. When the number of groups is too small (e.g., 2), the limited interaction between spectral bands may restrict the representation capability. Conversely, increasing the number of groups beyond 4 leads to a gradual decline in performance. This could be attributed to the fact that excessively large group numbers reduce the number of channels per group, potentially weakening the feature extraction capacity within each branch and making the network more susceptible to optimization difficulties. Furthermore, larger group numbers generally incur higher computational overheads during training. Therefore, a group size of 4 offers a balanced tradeoff between feature diversity and model efficiency for the tested datasets.

## 5. Conclusions

Due to the limited perception domain and sensitivity to the imbalance of spectral bands of Convolutional Neural Networks in hyperspectral image classification, CSFCNet has been proposed, which is a novel cascaded spatial-frequency convolutional network. First, the Dual Spatial-Fourier Convolution module effectively integrates hierarchical spatial details and directionally aware global textures. Second, the group-cascaded structure can achieve progressive semantic refinement between channel groups while reducing the number of model parameters. Finally, the Lightweight Local Attention module improves the discrimination of different land-cover categories without increasing a large amount of the computational overhead. Extensive experiments on three benchmark datasets demonstrate that CSFCNet achieves competitive classification accuracies compared to those of eight excellent methods, with comprehensive ablation studies validating the efficacy of each designed module.

## Figures and Tables

**Table 1:** Numbers of training and testing samples for the IP dataset.

No.	Color	Name	Train	Test
1		Alfalfa	3	43
2		Corn-notill	71	1357
3		Corn-mintill	41	789
4		Corn	13	224
5		Grass-pasture	24	459
6		Grass-trees	38	692
7		Grass-pasture-mowed	1	27
8		Hay-windrowed	25	453
9		Oats	1	19
10		Soybean-notill	50	922
11		Soybean-mintill	125	2330
12		Soybean-clean	30	563
13		Wheat	10	195
14		Woods	64	1201
15		Buildings-Grass-Trees-Drives	19	367
16		Stone-Steel-Towers	6	87
Total			521	9728

**Table 2:** Numbers of training and testing samples for the SA dataset.

No.	Color	Class	Train	Test
1		Broccoli-green-weeds_1	20	1989
2		Broccoli-green-weeds_2	37	3689
3		Fallow	20	1956
4		Fallow-rough-plow	14	1380
5		Fallow-smooth	27	2651
6		Stubble	39	3920
7		Celery	36	3543
8		Grapes-untrained	113	11,158
9		Soil-vineyard-develop	62	6141
10		Corn-senesced-green-weeds	33	3245
11		Lettuce-romaine-4wk	11	1057
12		Lettuce-romaine-5wk	19	1908
13		Lettuce-romaine-6wk	9	907
14		Lettuce-romaine-7wk	11	1059
15		Vineyard-untrained	72	7196
16		Vineyard-vertical-trellis	18	1789
Total			541	53,588

**Table 3:** Numbers of training and testing samples for the HH dataset.

Class	Color	Name	Train	Test
1		Red roof	140	13,901
2		Road	35	3477
3		Bare soil	218	21,603
4		Cotton	1632	161,653
5		Cotton firewood	62	6156
6		Rape	446	44,111
7		Chinese cabbage	241	23,862
8		Pakchoi	41	4013
9		Cabbage	108	10,711
10		Tuber mustard	124	12,270
11		*Brassica parachinensis*	108	10,905
12		*Brassica chinensis*	90	8864
13		Small *Brassica chinensis*	225	22,282
14		*Lactuca sativa*	74	7282
15		Celtuce	10	992
16		Film-covered lettuce	73	7189
17		Romaine lettuce	30	2980
18		Carrot	32	3185
19		White radish	87	8625
20		Garlic sprout	35	3451
21		Broad bean	13	1315
22		Tree	40	4000
Total			3866	382,827

**Table 4:** The detailed classification accuracies of IP on different networks. The best results are outlined in bold.

Class No.	SVM	HybridSN	3DCNN	GAHT	SSFTT	HyperFKAN	CLOLN	CSCANet	CSFCNet
1	71.10 ± 3.12	**83.09 ± 2.79**	56.82 ± 2.94	70.45 ± 1.38	61.40 ± 3.44	81.82 ± 8.34	82.60 ± 3.09	82.95 ± 4.62	77.45 ± 2.67
2	31.60 ± 3.41	88.87 ± 8.46	89.83 ± 2.29	**95.25 ± 2.14**	93.57 ± 1.30	91.83 ± 3.63	91.96 ± 3.24	94.92 ± 0.71	94.55 ± 0.59
3	19.83 ± 7.08	97.33 ± 9.08	90.62 ± 0.65	94.32 ± 1.62	97.03 ± 1.18	95.31 ± 2.20	95.51 ± 1.91	96.94 ± 0.97	**97.47 ± 1.44**
4	56.89 ± 6.93	**98.67 ± 1.04**	85.33 ± 1.01	92.36 ± 3.16	91.70 ± 3.93	91.47 ± 3.46	95.71 ± 4.46	92.76 ± 1.62	95.32 ± 1.52
5	21.75 ± 14.08	93.68 ± 3.56	77.78 ± 1.76	93.94 ± 3.41	99.32 ± 0.40	95.42 ± 0.73	93.83 ± 4.17	98.93 ± 0.86	**99.35 ± 0.18**
6	51.58 ± 5.67	97.26 ± 1.80	98.85 ± 0.63	97.78 ± 1.16	98.22 ± 0.89	99.22 ± 0.44	97.31 ± 2.67	**98.11 ± 0.49**	98.56 ± 0.87
7	76.72 ± 15.59	51.85 ± 6.66	44.44 ± 5.22	48.89 ± 19.67	40.74 ± 40.17	61.48 ± 17.49	28.73 ± 46.30	93.42 ± 4.45	**99.08 ± 1.85**
8	58.97 ± 11.83	98.12 ± 8.21	85.90 ± 1.18	99.87 ± 0.20	99.25 ± 0.80	99.96 ± 0.10	96.91 ± 4.69	99.98 ± 0.07	**100 ± 0.00**
9	56.39 ± 7.24	36.84 ± 2.22	84.21 ± 2.09	30.53 ± 20.18	45.26 ± 32.65	48.42 ± 7.45	46.00 ± 49.93	90.64 ± 3.85	**94.74 ± 4.30**
10	34.13 ± 7.05	88.95 ± 15.95	94.91 ± 7.62	95.36 ± 2.24	94.86 ± 1.93	94.14 ± 2.16	88.78 ± 6.71	**97.83 ± 0.61**	96.26 ± 1.52
11	54.78 ± 7.04	95.45 ± 3.25	94.77 ± 2.26	95.74 ± 1.98	97.44 ± 0.77	97.05 ± 0.71	94.87 ± 4.63	**98.64 ± 0.30**	98.53 ± 0.57
12	10.51 ± 7.41	81.71 ± 2.45	90.76 ± 6.67	91.30 ± 2.66	88.29 ± 1.58	**92.66 ± 5.37**	89.75 ± 7.52	92.62 ± 1.46	90.50 ± 1.93
13	85.64 ± 7.19	94.87 ± 1.94	79.49 ± 3.01	97.44 ± 1.81	97.84 ± 3.46	98.87 ± 1.05	95.90 ± 4.14	98.23 ± 1.09	**99.87 ± 0.25**
14	77.49 ± 10.02	98.34 ± 0.53	96.09 ± 1.92	98.84 ± 0.49	98.06 ± 0.91	99.53 ± 0.15	97.66 ± 1.64	99.40 ± 0.42	**99.44 ± 0.61**
15	18.45 ± 2.33	92.92 ± 1.36	76.02 ± 3.08	96.24 ± 4.34	91.69 ± 4.73	**97.55 ± 2.42**	93.38 ± 5.57	93.85 ± 2.33	94.68 ± 2.19
16	88.47 ± 2.19	**98.86 ± 0.78**	69.32 ± 4.14	91.82 ± 2.94	85.51 ± 7.09	90 ± 5.93	81.79 ± 12.24	87.99 ± 8.96	93.97 ± 3.44
OA (%)	45.15 ± 1.03	92.79 ± 0.88	90.97 ± 0.94	95.47 ± 0.39	95.58 ± 0.37	95.77 ± 0.87	93.74 ± 1.11	**97.17 ± 0.27**	97.09 ± 0.20
AA (%)	50.89 ± 1.63	86.86 ± 1.31	82.20 ± 2.05	86.88 ± 2.26	87.51 ± 6.37	89.67 ± 3.46	85.67 ± 5.66	94.83 ± 0.73	**95.61 ± 0.32**
κ×100	37.77 ± 1.00	91.77 ± 0.52	89.71 ± 1.38	94.83 ± 0.44	94.96 ± 0.43	95.18 ± 1.00	92.86 ± 1.28	96.77 ± 0.30	**96.68 ± 0.23**

**Table 5:** The detailed classification accuracies of SA on different networks. The best results are outlined in bold.

Class No.	SVM	3DCNN	HybridSN	GAHT	SSFTT	HyperFKAN	CLOLN	CSCANet	CSFCNet
1	96.57 ± 0.90	98.84 ± 2.16	99.23 ± 0.18	99.75 ± 0.96	98.82 ± 1.24	99.30 ± 1.26	99.85 ± 0.24	99.93 ± 0.20	**100 ± 0.00**
2	96.79 ± 0.71	99.89 ± 0.12	99.05 ± 0.26	99.88 ± 0.03	99.96 ± 0.06	100 ± 0	99.60 ± 0.24	100 ± 0.00	**100 ± 0.00**
3	64.43 ± 6.96	99.86 ± 0.20	99.14 ± 0.20	100 ± 0.01	99.55 ± 0.59	100 ± 0.00	97.66 ± 2.27	100 ± 0.00	**100 ± 0.00**
4	98.66 ± 0.36	98.48 ± 1.31	**100 ± 0.00**	97.68 ± 0.62	97.62 ± 1.35	99.33 ± 1.10	97.41 ± 1.98	99.16 ± 0.36	99.38 ± 0.30
5	96.86 ± 1.12	97.17 ± 1.34	**99.95 ± 0.03**	99.28 ± 8.35	97.11 ± 1.69	93.54 ± 5.78	97.77 ± 3.29	98.05 ± 1.55	96.58 ± 0.96
6	98.93 ± 0.59	99.45 ± 0.63	**100 ± 0.00**	99.80 ± 2.75	98.62 ± 2.42	100 ± 0.00	99.89 ± 0.11	99.98 ± 0.05	99.95 ± 0.08
7	98.97 ± 0.35	99.58 ± 0.50	97.95 ± 1.30	**99.96 ± 0.11**	99.03 ± 0.53	99.91 ± 4.97	99.82 ± 0.34	99.89 ± 0.11	99.95 ± 0.06
8	93.08 ± 4.68	96.13 ± 1.16	96.93 ± 1.23	**99.84 ± 1.09**	98.85 ± 0.61	92.90 ± 2.91	96.94 ± 2.97	98.96 ± 0.42	99.07 ± 0.36
9	98.66 ± 0.42	99.96 ± 0.08	99.77 ± 0.15	100 ± 2.88	99.75 ± 0.65	99.56 ± 4.14	99.32 ± 0.96	**100 ± 0.00**	**100 ± 0.00**
10	68.16 ± 3.16	98.57 ± 0.81	99.90 ± 0.06	99.81 ± 0.11	98.43 ± 1.02	97.11 ± 1.19	96.24 ± 2.98	99.27 ± 0.28	**99.81 ± 0.07**
11	10.89 ± 5.69	94.90 ± 4.14	99.35 ± 0.36	99.86 ± 1.54	96.74 ± 2.49	94.57 ± 5.50	99.64 ± 0.47	99.82 ± 0.12	**99.99 ± 0.01**
12	84.69 ± 11.38	97.94 ± 4.96	**99.99 ± 0.01**	99.84 ± 8.09	98.89 ± 0.48	99.17 ± 0.50	99.30 ± 0.23	99.34 ± 1.66	99.95 ± 0.08
13	93.52 ± 14.39	86.04 ± 16.64	99.83 ± 0.07	94.60 ± 4.15	87.44 ± 2.76	91.36 ± 2.82	99.00 ± 1.78	96.77 ± 1.45	96.66 ± 1.57
14	88.34 ± 1.75	96.20 ± 1.40	99.69 ± 0.24	90.75 ± 1.75	95.33 ± 3.60	98.81 ± 1.12	98.90 ± 0.95	98.50 ± 1.26	**99.75 ± 0.18**
15	24.25 ± 6.38	91.51 ± 1.30	**99.58 ± 0.00**	98.19 ± 6.78	97.19 ± 0.71	97.92 ± 6.88	91.63 ± 3.54	97.15 ± 0.81	97.59 ± 0.92
16	72.04 ± 3.20	95.71 ± 2.65	95.48 ± 0.61	99.78 ± 0.13	99.09 ± 0.82	99.98 ± 0.05	99.64 ± 0.70	99.46 ± 0.40	**99.83 ± 0.14**
OA (%)	79.23 ± 1.21	97.03 ± 0.70	98.62 ± 0.98	99.12 ± 0.66	98.39 ± 0.35	97.59 ± 0.58	97.32 ± 0.38	99.09 ± 0.15	**99.21 ± 0.07**
AA (%)	79.12 ± 1.89	96.89 ± 1.41	98.12 ± 1.14	98.70 ± 1.23	97.66 ± 0.47	98.60 ± 1.54	98.00 ± 0.38	99.13 ± 0.14	**99.27 ± 0.13**
κ×100	76.57 ± 1.42	96.69 ± 0.78	**99.61 ± 0.24**	99.24 ± 0.78	98.21 ± 0.39	97.31 ± 0.67	97.02 ± 0.42	98.99 ± 0.16	99.12 ± 0.08

**Table 6:** The detailed classification accuracies of HH on different networks. The best results are outlined in bold.

Class No.	SVM	3DCNN	HybridSN	GAHT	SSFTT	HyperFKAN	CLOLN	CSCANet	CSFCNet
1	72.51 ± 1.17	96.14 ± 1.49	97.88 ± 0.19	98.03 ± 0.42	97.28 ± 0.45	97.73 ± 0.18	97.52 ± 0.17	**98.31 ± 0.19**	98.18 ± 0.61
2	74.30 ± 5.06	66.31 ± 13.97	85.51 ± 3.32	87.14 ± 6.98	85.02 ± 2.86	87.39 ± 1.61	**96.86 ± 0.21**	89.23 ± 6.75	90.39 ± 1.32
3	75.39 ± 5.19	94.57 ± 0.52	97.28 ± 0.56	97.93 ± 0.86	96.59 ± 1.21	97.09 ± 0.39	94.79 ± 0.62	97.62 ± 0.60	**97.90 ± 0.35**
4	71.85 ± 2.05	99.58 ± 0.16	99.83 ± 0.05	99.93 ± 0.04	99.83 ± 0.10	99.84 ± 0.04	98.77 ± 0.62	**99.94 ± 0.03**	99.91 ± 0.03
5	70.95 ± 5.26	51.09 ± 10.14	90.10 ± 2.99	90.66 ± 1.17	90.97 ± 1.48	94.82 ± 1.73	89.74 ± 3.33	93.85 ± 1.24	**95.64 ± 0.96**
6	81.48 ± 0.62	95.67 ± 0.98	99.13 ± 0.44	**99.70 ± 0.14**	99.30 ± 0.32	99.39 ± 0.15	97.07 ± 0.77	99.53 ± 0.17	99.60 ± 0.09
7	48.55 ± 1.78	87.83 ± 4.09	96.34 ± 0.81	96.61 ± 0.60	94.96 ± 1.37	95.37 ± 0.44	**99.55 ± 0.15**	97.58 ± 0.51	96.93 ± 0.91
8	25.86 ± 3.49	39.54 ± 9.41	88.61 ± 1.99	92.75 ± 1.68	83.05 ± 7.64	91.26 ± 1.68	**95.65 ± 2.12**	89.63 ± 4.20	91.35 ± 1.05
9	84.60 ± 1.53	95.95 ± 1.72	99.14 ± 0.47	98.97 ± 0.37	98.61 ± 0.60	**99.48 ± 0.36**	99.02 ± 0.44	99.34 ± 0.26	99.25 ± 0.18
10	31.48 ± 4.55	76.54 ± 8.16	95.11 ± 2.09	97.03 ± 1.43	95.17 ± 1.32	94.61 ± 0.77	94.61 ± 1.14	**97.25 ± 0.46**	95.89 ± 0.97
11	29.35 ± 2.09	69.43 ± 9.08	93.12 ± 1.24	94.75 ± 0.51	91.51 ± 4.71	**95.82 ± 0.52**	91.18 ± 5.33	94.75 ± 0.82	95.21 ± 1.31
12	57.46 ± 4.59	52.11 ± 8.29	92.32 ± 1.42	92.86 ± 2.40	92.36 ± 3.04	93.51 ± 0.96	**98.41 ± 0.73**	93.05 ± 1.88	93.47 ± 1.32
13	42.46 ± 8.25	78.81 ± 5.28	94.29 ± 1.20	95.40 ± 0.99	90.98 ± 2.11	95.52 ± 0.74	**96.94 ± 1.73**	95.21 ± 0.99	94.82 ± 0.49
14	44.04 ± 8.26	75.38 ± 6.68	93.33 ± 2.21	95.55 ± 0.89	93.30 ± 1.50	**97.02 ± 1.16**	92.22 ± 2.09	96.40 ± 1.12	96.54 ± 0.50
15	65.41 ± 4.58	68.43 ± 18.29	87.29 ± 5.45	90.52 ± 2.25	90.95 ± 2.03	**95.97 ± 1.87**	91.84 ± 3.17	89.36 ± 2.64	93.72 ± 1.85
16	63.61 ± 5.67	91.07 ± 3.99	97.74 ± 0.71	98.36 ± 0.32	95.97 ± 2.84	98.29 ± 0.63	94.73 ± 0.85	99.07 ± 0.41	**99.07 ± 0.26**
17	61.44 ± 10.81	75.04 ± 13.09	97.25 ± 1.87	**99.09 ± 1.66**	96.60 ± 2.56	97.32 ± 0.56	97.38 ± 0.81	98.80 ± 0.77	98.34 ± 0.98
18	62.17 ± 2.54	68.57 ± 14.11	93.23 ± 1.82	95.42 ± 2.60	90.80 ± 1.43	**98.15 ± 0.96**	97.97 ± 0.75	95.99 ± 1.03	97.10 ± 0.67
19	63.58 ± 2.40	82.96 ± 4.75	93.93 ± 1.42	94.39 ± 1.08	93.49 ± 2.67	96.14 ± 0.70	93.56 ± 5.90	**96.73 ± 0.73**	95.61 ± 1.02
20	67.54 ± 5.94	48.53 ± 14.40	91.53 ± 1.91	92.15 ± 0.89	85.99 ± 3.94	95.10 ± 1.34	**95.93 ± 1.69**	92.44 ± 2.20	91.65 ± 2.10
21	55.83 ± 11.10	11.94 ± 6.83	88.75 ± 5.59	95.67 ± 1.81	91.89 ± 4.02	91.41 ± 1.26	91.02 ± 7.03	92.36 ± 5.27	**94.77 ± 2.78**
22	69.19 ± 4.23	67.88 ± 20.21	96.09 ± 2.31	97.35 ± 2.00	94.02 ± 3.00	97.01 ± 1.50	95.85 ± 3.43	97.34 ± 1.55	**97.92 ± 1.64**
OA (%)	65.89 ± 1.03	89.65 ± 2.36	97.50 ± 0.24	98.06 ± 0.12	96.95 ± 0.10	95.87 ± 0.12	95.43 ± 3.00	**98.22 ± 0.26**	98.20 ± 0.07
AA (%)	59.96 ± 0.96	72.43 ± 5.91	93.99 ± 0.84	95.47 ± 0.49	93.12 ± 0.65	97.48 ± 0.25	83.78 ± 7.86	95.63 ± 1.01	**96.06 ± 0.27**
κ×100	65.91 ± 1.37	86.84 ± 3.02	96.84 ± 0.30	97.55 ± 0.15	96.15 ± 0.13	97.85 ± 0.15	95.81 ± 1.90	**97.75 ± 0.33**	97.72 ± 0.09

**Table 7:** Ablation study of the key components of our method: DSF-Conv module, group cascade structure, and LLA module. The best results are outlined in bold.

DSF-Conv	Group Cascade	LLA	Metric			
			IP	SA	HongHu	Paras
✓	✓	✓	**97.09%**	**99.21%**	**98.20%**	**328 k**
	✓	✓	92.71%	95.09%	95.36%	836 k
		✓	87.38%	96.12%	93.88%	210 k
✓		✓	96.23%	99.06%	97.55%	1204 k
✓	✓		95.19%	99.04%	98.18%	275 k

**Table 8:** Effects of PCA on the IP and SA datasets. The best results are outlined in bold.

Metric	IP		SA	
	No PCA	PCA_30_	No PCA	PCA_30_
OA (%)	93.44 ± 1.87	**97.09 ± 0.20**	93.04 ± 4.04	**99.21 ± 0.07**
AA (%)	85.61 ± 4.23	**95.61 ± 0.32**	82.67 ± 3.82	**99.27 ± 0.13**
κ×100	90.14 ± 2.93	**96.68 ± 0.23**	92.09 ± 4.51	**99.12 ± 0.08**

**Table 9:** Effects of different numbers of groups on the IP dataset. The best results are outlined in bold.

Metric	The Number of Different Groups			
	2	4	8	16
OA (%)	95.33 ± 2.91	**97.09 ± 0.24**	97.06 ± 0.56	95.67 ± 0.93
AA (%)	93.67 ± 4.63	**96.63 ± 0.87**	94.92 ± 0.58	90.13 ± 5.23
κ×100	95.14 ± 3.25	**97.23 ± 0.27**	96.71 ± 0.45	95.04 ± 1.47

## Data Availability

The data presented in this study are openly available in public repositories.
